# Accessible mental well-being intervention for adolescents in school settings: a single-group intervention study using a pretest–post-test design

**DOI:** 10.1186/s13034-023-00576-0

**Published:** 2023-02-20

**Authors:** Anna Tornivuori, Kim Kronström, Minna Aromaa, Sanna Salanterä, Max Karukivi

**Affiliations:** 1 Department of Nursing Science, University of Turku, Turku, Finland; 2 Department of Adolescent Psychiatry, University of Turku and Turku University Hospital, Turku, Finland; 3 Outpatient Clinic for Children and Adolescents, Turku University Hospital, Turku, Finland; 4 Turku University Hospital, Administration, Turku, Finland; 5 Psychiatric Care Division, Satakunta Hospital District, Pori, Finland; 6 Outpatient Clinic for Children and Adolescents, Turku University Hospital, Turku, Finland

**Keywords:** Adolescents, Anxiety, Cognitive-behavioral therapy (CBT), Depression, Intervention, Mental health, Mental disorders, Psychiatry, School, School nurses

## Abstract

**Background:**

A growing number of adolescents seek treatment for mental health problems, a circumstance that stresses the importance of implementing accessible treatment options. This study evaluates the impacts of brief, mental well-being intervention for adolescents in a school environment. As mental health interventions are often targeted at specific disorders, we sought a comprehensive approach to reach adolescents with a range of mental health symptoms.

**Methods:**

Single-group intervention study with a pretest–posttest design was utilized and conducted in lower, upper secondary, and vocational schools on adolescents ages 12–18 who sought medical attention for mental health symptoms. The cut-off point for inclusion was ≥ 14, for the Young Persons Clinical Outcomes for routine Evaluation (YP-CORE) measurement. The intervention included six face-to-face visits implemented by psychiatric nurses who received a 3-day training course. The impacts were evaluated after 6 weeks (*n* = 87) and again at 6 months (*n* = 68) and assessed using the YP-CORE, Beck Depression Inventory (BDI-II) and Overall Anxiety Severity and Impairment Scale (OASIS).

**Results:**

The participants reported significant levels of mental distress at baseline with a YP-CORE mean score = 21.48, a BDI-II mean score = 23.60, OASIS mean score = 10.98. Post-intervention results at 6 weeks for the primary outcome YP-CORE showed a significant (*p* < .001) mean score decrease of − 3.82, a medium effect size *d* = .627. For participants attending upper secondary and vocational schools the YP-CORE scores changed significantly from baseline to 6-weeks (*p* = .005) and from baseline to 6-months (*p* < .001). Long-term outcomes at 6-months showed a − 1.14 decrease (*p* = non-significant), effect size *d* = .175. After the 6-week intervention, 12% of the participants were assessed as not requiring additional visits.

**Conclusions:**

This easily accessible intervention in a school setting indicated improvement for those participants with mild to moderate mental disorder symptoms and attending upper secondary and vocational schools. After the 6-week intervention, significant positive effects were observed. Participants reported substantial levels of mental distress at the baseline, which could contribute to the decline of symptoms and need for extended care during the 6 months follow-up.

*Trial registration* Retrospectively registered with Clinicaltrials.gov identifier NCT05356949

**Supplementary Information:**

The online version contains supplementary material available at 10.1186/s13034-023-00576-0.

## Background

The prevalence of mental health disorders and related symptoms, such as anxiety and varying degrees of depression, increases during adolescence. Around 20% of adolescents suffer from mental health disorders [1] and these disorders are associated with a high prevalence of emotional and psychiatric problems in adulthood [2]. Adolescents' mental health disorders also pose a risk of their exclusion from society and can, therefore, cause significant educational and economic consequences [3–5]. The use of adolescent mental health services has thus grown steadily over the past decade [6]. In 2020, 14.4% of Finnish adolescents visited mental health services [7]. It has been speculated whether the use of adolescent mental health services has increased as the stigma on mental health has decreased and the threshold of seeking help has become lower. Identification of disorders in school mental health and primary healthcare services is to date more active, for example, due to these structural changes [8]. However, easily accessible care services have not developed at the same pace due to human resources issues and limited treatment methods available in local youth services [1].

Effective and easily accessible treatments are needed to support the mental well-being of children and adolescents. Some interventions have been developed for the treatment of mental disorders in local youth settings using methods that are based on cognitive behavioral therapy (CBT) that is implemented as short-term therapy [9] and also interpersonal therapy (IPT) and interpersonal counselling (IPC). All have been found to be effective in the school environment [10, 11]. At-risk adolescents benefit from early individually aimed interventions, as they may help these individuals to avoid the development of high mental and social burdens later in life [12, 13].

The severity of depression and other mental disorders can be viewed as a continuum. Mild and moderate forms of depression are characterized by a lesser number and severity of individual symptoms, and better general functioning. Briefly structured individually based interventions in school settings have been shown to be effective when treating mild to moderate depression [14, 15]. Nearly all adolescents are also in contact with health care professionals in school settings, making the route an easily accessible entry to receive mental health services [16] and a fruitful environment for facilitating different stakeholders to work together [17]. In Finland, health care in schools is a free statutory primary health service. A full-time school nurse on average is responsible for 600 pupils [18, 19]. In an initiative to enhance mental support for adolescents, the City of Turku placed ten psychiatric nurses in schools at the end of 2019. The psychiatric nurses work in collaboration with school nurses aiding the identification and support of mild to moderate mental disorder symptoms.

The emerging research shows promising results for mental health school-based interventions for alternative treatment providers for easy accessibility, low disruption to schoolwork, increased treatment, and long-lasting improvements [20–22]. Due to the easy accessibility of school nurses and school being part of an adolescent’s everyday life, the school environment could be a beneficial venue for reaching reluctant and unresponsive adolescents [23]. Despite there still being some challenges when providing mental health services in schools, such as the identification of specific mental health problems and achieving adequate staffing, the positive outcomes of school-based interventions still prevail [24]. However, there are substantial gaps in the resources currently available for child and adolescent mental health services and thus a need for innovative training approaches [1].

There is also a shortage of research with long-term follow-up on the cost-effectiveness of mental health interventions. The use of resources in mental health interventions varies greatly based on the settings, use of professionals, delivery of the intervention (group, individual, or a combination) intensity, and duration and availability, all of which impact the overall cost-effectiveness of delivered interventions [25]. Mental health interventions are often targeted at specific disorders, such as depressive disorders, using cognitive-behavioral therapy that may be timely and demand financial resources [1]. There is thus a great demand for innovative easy-to-implement methods to improve the accessibility of effective mental health services for adolescents [15]. Mental health interventions delivered by health-care workers in the school environment are still scarce [26]. However, based on previous research, good short-term results may still be achieved using different approaches [17].

The aim of this study was to evaluate the impacts of a brief and structured intervention in school settings for adolescents with various mild to moderate mental disorder symptoms, implemented by psychiatric nurses working in collaboration with school nurses. Considering the shortage of studies that have assessed long-term results, in addition to short-term results, we aimed to assess the results after 6-months of follow-up. We also did not set limitations on the mental disorder symptoms (anxiety, depression etc.) the adolescent reported seeking help for, so as to learn the effectiveness of the intervention process on overall common mental disorders symptoms. We hypothesized that this studied intervention would result in achieving similar results in the short term as the previously researched brief interventions in the school environment.

## Methods

### Participants

Adolescents ages 12–18, from lower secondary, upper secondary, vocational schools, and others who looked for medical attention due to a mental health symptom or who had received a mental health diagnosis at school or via college health screenings were eligible for this study. The study was conducted in Turku, Finland. Inclusion criteria was having ≥ 14 pts in Young Persons Clinical outcomes for the routine Evaluation (YP-CORE) measure that measures overall mental well-being [27]. We defined substance abuse problems, already existing mental health service contact and insufficient knowledge of the Finnish language as the exclusion criteria. The excluded adolescents were treated using the standard treatment pathways. Based on a calculation of the original sample size, the target was to recruit both an intervention group (n = 300) and a treatment-as-usual control group (n = 300). However, the COVID-19 pandemic and its related restrictions started soon after recruitment initiation for the study and severely hindered it. The restrictions made it impossible to recruit the control group, and the target size for the intervention group had to be lowered to 150.

Written informed consent was requested from all the study participants. Additional parental permission to participate was sought for subjects under the age of 15. Adolescents were informed of the voluntary nature of their participation in the study and the choice of suspending or withdrawing their participation at any stage. Failure to participate in the study would not affect their treatment in any way. If necessary, a psychiatric nurse would consult with an adolescent psychiatrist during the intervention.

### The intervention

This single-group intervention study was implemented by psychiatric nurses who had received a three-day method training based on the tools and theory of cognitive behavioral therapy. The psychiatric nurses were taught exposure, behavioral activation, problem-solving, and relaxation techniques and also received training on the measures being used in the study. Since the core concept was that the intervention was not tied to any certain disorder or symptom, the intervention was not manualized. The psychiatric nurses could independently choose from the given tools, what to use depending on the situation, and the needs of the participating adolescent. The training was also conducted by an experienced psychotherapy trainer.

The intervention was conducted in lower secondary, upper secondary, and vocational schools, or other and included six face-to-face visits of 45 minutes. On the first visit, the adolescents’ mental well-being and ability to function were assessed using measures of the YP-CORE [27, 28] Beck Depression Inventory, BDI [29], and Overall Anxiety Severity and Impairment Scale, OASIS [31]. The adolescents also defined their personal goals, and then together with the psychiatric nurse, considered how to achieve those goals. The adolescents were supported in changing their thinking, emotional life, and behavior activation using cognitive behavioral therapy methods, endorsed by conducting related homework. At 6 weeks, post-intervention assessments were conducted during the visit. At 6 months, a research assistant contacted the adolescents via mobile phone/text message. For those willing to participate, a link to the questionnaires was sent. If the adolescents did not respond to that first contact, a follow-up call was made. All assessments throughout the intervention were self-reported and the psychiatric nurses saved all research data to the REDCap tool. The low threshold for the adolescents to seek help, the straightforward implementation, and basic training that did not require large resources were the basis for achieving easy accessibility of the intervention.

Data collection started in January 2020, but the collection had to be suspended in March 2020 due to the COVID-19 pandemic and its related restrictions. Recruitment was resumed in August 2020. The recruitment of adolescents lasted until the end of November 2021 and the 6-month follow-up ended in May 2022. The TIDier checklist was used to improve the description of the intervention (Additional file 1) [32].

### Baseline characteristics

Self-reported data were collected from the participants regarding their personal characteristics at baseline for age, gender (boy/girl/other), type of school (lower secondary school/upper secondary school/vocational school or other), living conditions (single parent home/both parents/other), parental unemployment during previous year (one parent/both parents), self-reported reason for seeking help (anxiety/depression/other) treatment for mental health, (mental health treatment/usage of mental health medication).

### Measurements

The YP-CORE is a measure of overall psychological well-being, and it is used to measure the response to psychotherapy. It consists of 10 questions and gives scores between 0 to 40. A higher score indicates lower well-being. The cutoff point of ≥ 14 may be used to distinguish significantly symptomatic persons [27] and that point was used as an inclusion criterion in the present study. The score was measured at baseline, at 6-week and 6-month time-points and as a continuous variable in this study. The main response variable was the change in YP-CORE scores during the intervention. The psychometric properties were further evaluated to be clear for the Finnish translation [28].

BDI-II a 21-item questionnaire used to assess depressive symptoms [29]. The score range for this measure is 0–63. The measure has been widely used and its psychometric properties have been evaluated to work well also for the Finnish translation [30]. The score was measured at baseline, at 6-week, and 6-month time-points, and used as a continuous variable in this study.

The Overall Anxiety Severity and Impairment Scale (OASIS) is a short 5-point measure used to assess the severity of anxiety and dysfunction due to anxiety [31]. The questions are scored 0–4, so the total score ranges from 0 to 20. We used these scores as continuous variables with the same measuring points as YP-CORE and BDI-II uses.

The participants answered an open-ended question on the primary reason why they were seeking help. Most of the participants reported anxiety or depression, but some reported another reason (for example, difficulties sleeping or interpersonal problems). Based on these answers, the participants were categorized into three groups, namely, (Anxiety/Depression/Other).

After the intervention, the nurses assessed whether a participant needed additional visits (none/one to four visits/five or more visits). Additionally, at the 6-month follow-up, the participants reported on whether they had received treatment other than the treatment offered in the study intervention during the follow-up (yes/no) and, if they had received that treatment, from where (health care center/specialized mental health care/other).

### Statistics

The normality of the distributions of the variables was assessed both graphically and via the Shapiro–Wilk Test. Due to their parametric distributions, the YP-CORE, BDI, and OASIS scores are presented as the means with 95% confidence intervals (95% CI). Age is characterized using medians and interquartile range (IQR), since its distributions were positively skewed.

For the attrition analyses from baseline to 6-week follow-up, categorized variables were compared for the participants and the dropouts using the Chi-Square test. For the continuous variables, the differences were analyzed using the t-test for normally distributed variables and the Mann–Whitney U Test for non-normally distributed variables.

The changes in the YP-CORE, BDI and OASIS scores from the baseline to the 6-week follow-up and the 6-month follow-up were assessed using paired samples t-tests. The effect size for the mean score difference was estimated using Cohen’s *d* [33]. Cohen’s *d* of 0.2 represents small, 0.5 represents medium, and 0.8 indicates large effects. The analyses were conducted for the sample as a whole; however, additional group-wise analyses were also conducted.

At all the time-points, the association of the continuous variables with the YP-CORE, BDI-II and OASIS scores was assessed using the Spearman correlation for age and the Pearson correlation for the parametrically distributed variables. For the categorized variables, a t-test was used for variables with two categories, and ANOVA was used for variables with three or more categories.

To control for the effects of background variables and group differences, the 6-week outcomes were analyzed using ANCOVA. In these ANCOVA analyses, age, gender, and the corresponding baseline score were used as co-variates. In these analyses, gender was used as a dichotomized variable (girl/boy or other). Additionally, the analyses included variables that were significantly (p < 0.05) associated with the respective outcome scores (YP-CORE, BDI-II, or OASIS) in the univariate analyses. The fit of the models was confirmed based on the normality and variance of the residuals.

Repeated measured scores for YP-CORE, BDI-II, and OASIS were analyzed using a linear mixed model with the patient indicator as a random effect and gender, school, time, gender-by-time, and school level-by-time as fixed effects. When the interaction effect showed statistical significance, the contrasts were programmed to solve and indicate where that significance difference occurred. An optimal covariance structure was chosen based on the data (either unstructured or compound symmetry). The Kenward-Roger correction for degrees of freedom was used.

The internal consistency of the YP-CORE, BDI-II and OASIS measurements were calculated at the baseline using Cronbach’s alpha. The reliability of the YP-CORE questionnaire was found to be good (*a* = 0.603). The BDI-II was highly reliable (*a* = 0.877) and was similarly so for the OASIS scale (*a* = 0.795). In all the analyses, the *p*-values < 0.05 were considered to be statistically significant. Statistical analyses were carried out using the IBM SPSS software, Version 270. The linear mixed models were generated using SAS software, Version 9.4 of the SAS System for Windows (SAS Institute Inc., Cary, NC, USA).

## Results

**Fig. 1:**
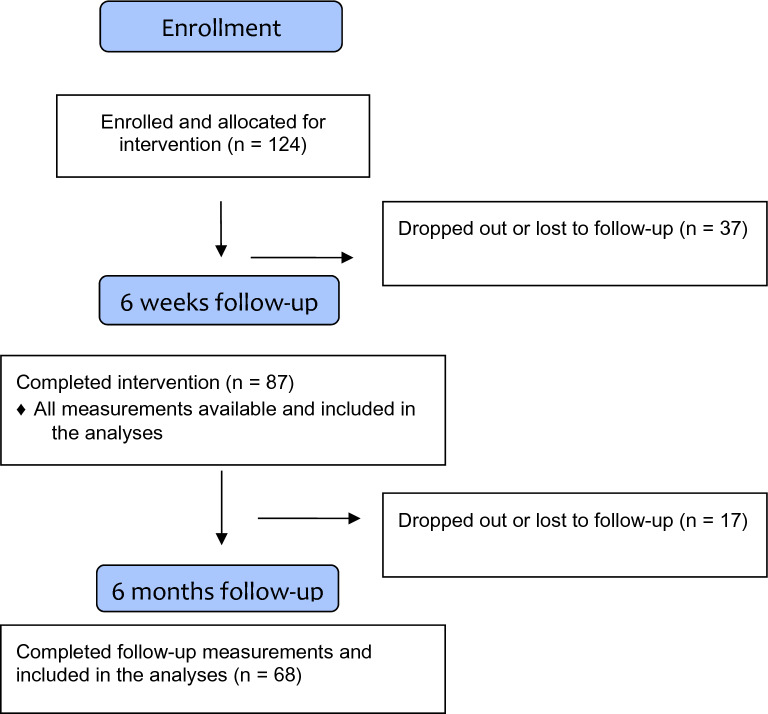
Flow chart of participants in the study

Initially, 124 adolescents were recruited, but for this study, only those participants who took part in the post-intervention assessments were included *(n* = 87). A flow chart for the participants is shown in Fig. 1. The baseline characteristics of the participants are presented in Table 1. The age of the participants ranged from 13 years to 19, fathered from a school distribution of lower secondary (*n* = 32), upper secondary (*n* = 40), vocational schools (*n* = 13) and other (*n* = 2). There were more girls who participated than boys. There were also no statistically significant differences regarding the outcome between girls and boys. Thus, the sample was analyzed as a whole.

**Table 1:** Characteristics of the study participants

Variable	n (%)	YP-CORE, Baseline	n	BDI-II, Baseline	n	OASIS, Baseline
		Mean (95% CI)		Mean (95% CI)		Mean (95% CI)
Gender						
Girl	76 (87.4)	21.71 (20.56–22.86)	72	22.14 (22.14–26.28)	71	11.25 (10.50–12.01)
Boy	8 (9.2)	19.00 (16.36–21.64)	8	16.88 (12.12–21.63)	8	8.62 (6.22–11.03)
Other	3 (3.4)	22.33 (14.47–29.92)	2	28.50 (-3.27–60.27)	2	10.50 (4.14–16.85)
Total	87 (100)		83		83	
P-value^a^		0.31		0.06		0.08
School where studying						
Lower secondary school	32 (36.8)	21.66 (19.83–23.48)	30	25.07 (21.32–28.81)	29	11.21 (10.02–12.39)
Upper secondary school	40 46.0)	21.75 (20.48- 23.02)	39	22.69 (20.27–25.11)	39	10.36 (9.28–11.43)
Vocational school or other	15 (2.3)	20.40 (16.76–240.4)	13	22.92 (17.24.-28.61)	13	12.30 (10.70–13.91)
Total participants	87 (100)					
P-value^a^		0.64		0.52		0.14
Living conditions						
With both parents, all in the same house	45 (51.7)	20.89 (19.65–22.13)	42	22.62 (19.94–25.29)	42	10.69 (9.46–11.74)
Mostly with one parent	29 (33.3)	21.83 (19.73–23.92)	27	24.33 (20.45–28.22)	27	11.38 (10.01–12-76)
Other	13 (14.9)	22.77 (19.31–26.23)	13	25.23 (21.02–29.44)	13	11.08 (9.80–12.35)
Total	87 (100)					
P-value^a^		0.46		0.77		0.94
Parent unemployment (last year) one of their parents						
Yes	24 (27.6)	21.88 (20.03–23.72)	23	25.35 (21.95–28.749	22	11.36 (9.96–12.76)
No	61 (70.1)	21.49 (20.19–22.79)	57	22.84 (20.41–25.28)	57	10.86 (10.0–11-729
Total	85 (97.7)		80		79	
P-value^b^		0.38		0.32		0.74
Self-reported reason for seeking help						
Anxiety	36 (41.4)	20.31 (18.83–21.78)	35	20.77 (17.87–23.67)	35	11.57 (10.73–12.41)
Depression	18 (20.7)	22.50 (20.06–24.94)	18	26.22 (21.80–30.65)	17	11.41 (9.84–12.98)
Other	23 (88.5)	21.74 (19.36–24.12)	21	25.05 (21.07–29.02)	21	9.42 (7.73–11.12)
Total	77 (88.5)		74		73	
P-value^a^		0.25		0.06		0.03
Have you received treatment for mental health problems in the last year?						
Yes	35 (40.2)	21.77 (19.93–23.62)	34	24.47 (21.07- 27.87)	34	11.06 (9.71–12.41)
No	50 (57.5)	21.16 (19.86–22.46)	47	22.81 (20.45–25.16)	46	10.93 (10.12–11.70)
Total	85 (97.7)		81		80	
P-value^b^		0.21		0.19		0.009 = 0.01
Have you been taking medication for mental health problems?						
Yes	8 (9.2)	21.63 (16.75–26.50)	7	25.14 (14.29–36.00)	7	11.43 (8.71–14.14)
No	79 (90.8)	21.47 (20.39–22.54)	75	23.45 (21.50–25.41)	74	10.93 (10.18–11.68)
Total	87 (100)		82		81	
P-value^b^		0.59		0.25		0.60

^a^One-way ANOVA

^b^T-test

For the attrition analysis, we compared the characteristics at baseline, 6 weeks, and 6 months. The results of participant loss at 6 weeks showed random loss in all categories other than age. The dropouts were older (mean = 17.0 years, *SD* 1.22) than the participants still attending (mean = 16.1 years, *SD* = 1.50), *p* < 0.01. Also at 6 months, those who had dropped out were older than the participants, but that difference was not statistically significant.

### Primary and secondary outcome measures

The adolescents reported relatively high mean scores at the baseline measured by YP-CORE (mean = 28.58), BDI-II (mean = 23.60) and OASIS (mean = 10.98). The mean scores for the primary outcome measures (YP-CORE) and the secondary outcome measures (BDI-II and OASIS) decreased immediately after the intervention at 6 weeks, but then slowly increased in the 6 months follow-up. The changes in the total scores at 6 weeks for YP-CORE showed a mean score decrease of − 3.82 and a medium effect size *d* = 0.63. Also, for the BDI-II and the OASIS scores, statistically significant decreases were indicated at 6 weeks. See Table 2.

**Table 2:** Measurements of the participants’ mental well-being at baseline, 6-weeks, and the 6-months follow-up

	*N*	Total Scores	Change	Effect Size	*(p)*
		Mean (*SD*)	Mean	*Cohen’s d*	
YP-CORE					
Baseline	87	21.48 (4.86)			
6 weeks	87	17.67 (7.27)	− 3.82	0.63	< 0.001
Baseline	69	21.20 (4.48)			
6 months	69	20.06 (6.74)	− 1.14	0.17	0.904
BDI − II					
Baseline	82	23.60 (8.75)			
6 weeks	82	19.32 (11.01)	− 4.28	0.52	< 0.001
Baseline	63	22.95 (8.24)			
6 months	62	21.94 (12.51)	− 0.76	0.07	0.896
OASIS					
Baseline	81	10.98 (3.19)			
6 weeks	81	9.62 (3.55)	− 1.36	0.49	< 0.001
Baseline	62	10.97 (3.25)			
6 months	62	10.61 (4.01)	− 0.35	0.11	0.052

YP-CORE = The Young Persons Clinical outcomes for routine Evaluation, BDI-II = Beck Depression Inventory, OASIS = The Overall Anxiety Severity and Impairment Scale

For the outcome scores at 6 weeks, only the school where the participant was studying was significantly (p = 0.024) associated with the YP-CORE scores, while other background variables or previous treatment were not. The school where the participant was studying was also significantly (p = 0.003) associated with the BDI-II scores, and previous treatment during the last year with the OASIS scores (p = 0.033). In the ANCOVA analyses that were controlled for age, gender and corresponding baseline scores, the baseline YP-CORE score (p < 0.001) and age (p = 0.001) were significantly associated with the score at 6 weeks. The adjusted R^2^ was 0.376. For the BDI-II score at 6 weeks, only the BDI-II baseline score was significantly (p < 0.001) associated, and the adjusted R^2^ was 0.545. For the 6-week OASIS scores, both the baseline score (p < 0.001) and age (p = 0.010) were significantly associated (Adjusted R^2^ = 0.495).

For the YP-CORE scores, the mean changes from baseline to 6 months were not statistically significantly different between girls and boys (p = 0.38). However, the changes were significantly different for the schools where the participants studied p = 0.006). Lower secondary school students differed significantly from upper secondary school students both between the baseline and 6 weeks (p = 0.005) and the baseline and 6 months (p < 0.001). While the YP-CORE scores decreased for upper secondary and vocational school students during the intervention and were at a lower level also at the six- month follow-up, these same scores did not significantly change for lower secondary school students (Table 3).

**Table 3:** Model-based means for the group using time-repeated measures analyses

Dependent	Effect	Group	Timepoint	Estimate	95% *CI*	*P*^a^
YP-CORE	School x time	Lower secondary school	Baseline	20.86	18.26–23.44	< 0.001
			6 weeks	19.25	16.67–21.84	< 0.001
			6 months	22.19	19.31–25.08	< 0.001
		Upper secondary school	Baseline	21.05	18.76–23.35	< 0.001
			6 weeks	15.32	13.03–17.61	< 0.001
			6 months	16.71	14.21–19.20	< 0.001
		Vocational school	Baseline	19.33	15.65–23.00	< 0.001
			6 weeks	16.34	12.67–20.02	< 0.001
			6 months	17.43	13.24–21.63	< 0.001
BDI-II	School x time	Lower secondary school	Baseline	23.00	19.18–26.82	< 0.001
			6 weeks	23.48	19.13–27.84	< 0.001
			6 months	23.50	17.93–29.06	< 0.001
		Upper secondary school	Baseline	21.13	17.82–24.43	< 0.001
			6 weeks	16.00	12.13–19.85	< 0.001
			6 months	16.45	11.59–21.31	< 0.001
		Vocational school	Baseline	21.23	15.79–26.67	< 0.001
			6 weeks	14.56	8.37–20.75	< 0.001
			6 months	14.48	6.42–22.53	< 0.001
OASIS	School x time	Lower secondary school	Baseline	10.11	8.73–11.49	< 0.001
			6 weeks	8.93	7.46–10.39	< 0.001
			6 months	9.53	7.76–11.30	< 0.001
		Upper secondary school	Baseline	9.63	8.44–10.82	< 0.001
			6 weeks	7.99	6.67–9.31	< 0.001
			6 months	8.25	6.70–9.80	< 0.001
		Vocational school	Baseline	11.26	9.30–13.22	< 0.001
			6 weeks	7.19	5.11–9.27	< 0.001
			6 months	7.53	4.97–10.09	< 0.001

YP-CORE = The Young Persons Clinical outcomes for routine Evaluation, BDI-II = Beck Depression Inventory, OASIS = The Overall Anxiety Severity and Impairment Scale, 95% CI = 95% Confidence Interval

^a^Linear mixed model

For the BDI scores, gender did not have a statistically significant effect on the mean change from baseline to 6 months (p = 0.15), but the effect of school was significant (p = 0.019). From baseline to 6 weeks, lower secondary school students differed significantly from both upper secondary school students (p = 0.003) and vocational school students (p = 0.005). The differences from baseline to 6 weeks and from 6 weeks to 6 months were not statistically significant. The model-based means, together with 95% CI, are presented in Table 3.

Again, for the OASIS scores, the mean changes from baseline to 6 months did not statistically significantly differ by gender (p = 0.25), but the changes were significantly different by school (p = 0.026). The lower secondary school students differed significantly from the vocational school students both from baseline to 6 weeks (p = 0.001) and from baseline to 6 months (p = 0.006), but not from 6 weeks to 6 months (p = 0.80). The differences between the lower secondary school students and upper secondary school students were not significant, but the differences for the upper secondary school students did differ significantly for vocational school students from baseline to 6 weeks (p = 0.005) (Table 3).

### Comparisons based on self-reported reasons for seeking help

**Fig. 2:**
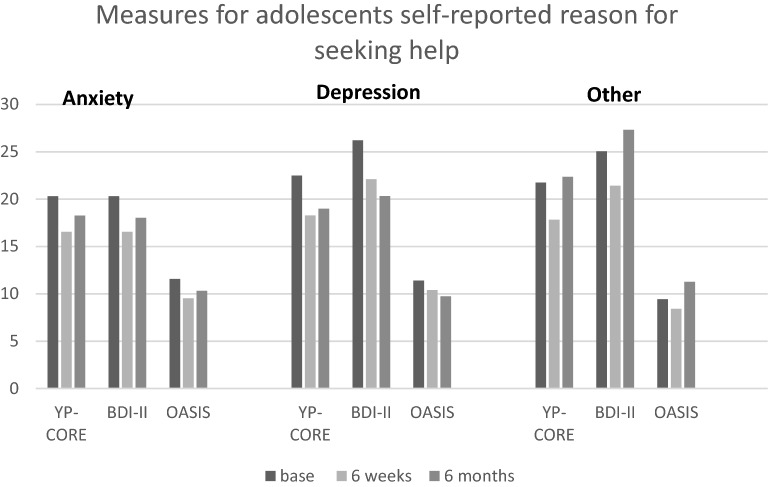
Measures for adolescents self-reported reason for seeking help. Comparisons between the different categories based on the participants’ self-reported reasons for seeking help at baseline, 6 weeks, and 6 months. YP-CORE, The Young Persons Clinical outcomes for routine Evaluation; BDI-II, Beck Depression Inventory; OASIS, The Overall Anxiety Severity and Impairment Scale

Participants with anxiety, depression, or other reasons for seeking help showed the same decreasing trend in scores at the 6 weeks’ follow-up as for the primary outcomes. Throughout the whole intervention, at all the time measuring points, the most enduring decrease in scores was seen for the depressive participants. At the 6 weeks’ follow-up, the biggest decrease in the mean scores was seen for anxiety symptoms; however, at the 6 months’ follow-up, the change in the mean scores was moderate. For those participants’ seeking help for other reasons, there was a similar decreasing trend at 6 weeks, but those results did not hold at the 6 months’ follow-up. See Fig. 2.

At 6 weeks, the psychiatric nurses evaluated and determined that only a minority 12% (*n* = 11) of the participants would not require any additional visits with them. Altogether 44% (*n* = 38) were estimated to require one to four visits, and 43% (*n* = 37) would need five or more visits. At 6 months’ follow-up, the majority at 62% (*n* = 54) of the participants reported to have received some other treatment than the study intervention during the follow-up. These participants reported receiving additional help from health care centers 5% (*n* = 4), specialized mental health care 23% (*n* = 20) or other professionals 35% (*n* = 30). Additional visits or receiving other treatment during the follow-up were not statistically significantly associated with the YP-CORE, BDI-II, or OASIS scores at the 6 months follow-up.

## Discussion

The study shows that this studied, but brief, mental well-being intervention in school settings was beneficial for adolescents attending upper secondary or vocational schools who had a range of different psychological symptoms. The short-term results showed a significant decrease in the measures of overall psychological well-being, as well as the depressive and anxiety symptoms, irrespective of the student’s self-reported reasons for seeking help (anxiety, depression or other reasons). These results are in line with previous research that shows that school-based mental health interventions are applicable and also effective for common mental disorders that are prominent in the adolescent population [14, 34].

The short-term results showed significant symptom reduction with its measures on overall psychological well-being, depressive symptoms, and the severity of anxiety for the participants attending upper secondary or vocational schools. Based on these effect sizes, the results were in the same range comparably, for example, with a previous school-based intervention study with IPC [15]. Interestingly, these results are also similar when compared with an earlier study conducted with the same YP-CORE translation among Finnish adolescents who were attending specialized outpatient care for 3 months [28]. The present results were similar, regardless of the reason for seeking help, namely, anxiety, depression or another, indicating short-term benefits for a wide range of mental health symptoms. Interestingly, this intervention was most beneficial for those participants that had reported depression or anxiety as reasons for seeking help, since the follow-up scores did not rise to the baseline levels. For those participants who had reported other reasons for seeking help, the scores here at 6 months follow-up were higher than at baseline. This result indicates that the intervention was not sufficient for this group, and there may need to be further specific actions to target these symptoms adequately. Previously, the research commonly has focused on specific disorders, such as, for example, interpersonal counseling (IPC) for depressive symptoms [11, 12]. The results of this current study support the idea that easily accessible mental interventions with a more general approach to address a wider range of mental health symptoms may be feasible in school settings.

The positive results achieved here for overall psychological well-being scores improved for those participants who were attending upper secondary school or vocational schools. The measures used are suitable for younger adolescents and do not explain this result [27, 28]. Compared with the previously suggested clinically meaningful changes for the YP-CORE scores [35], the change scores in the present study were smaller. However, since there are not yet fully comparable scores for the Finnish population, these comparisons should be made cautiously. It is also noteworthy that the score changes that Twigg et al. reported responded to effect sizes that are markedly higher than those for psychotherapy interventions on average [36]. However, there may also have been differences in the implementation methods used for the intervention, for example, the younger participants’ ability to benefit from the received treatment or factors that related to the individual psychiatric nurses delivering the interventions. Different psychiatric nurses worked in lower secondary and upper secondary schools; still, given the scope of this study, we were not able to separate these elements. Further studies are still needed to investigate how differences in implementation of interventions affected the results.

The lack of long-term benefits from this study is consistent with the findings from previous studies [16, 37]. However, long-term outcomes are still mostly unknown due to a scarcity of studies that have long term follow-ups [26, 37, 38]. The effects of psychotherapy on depression seem to improve with and an increase in the age of those receiving interventions, from children to adolescents and young adults [36]. It is possible that a similar age-effect may play a role in the effectiveness of the mental health intervention that was used in the present study. This possibility is supported by our results, which suggest that older participants benefited more from the intervention than did younger ones. For this particular intervention, the YP-CORE cut-off point was set at 14, which indicates on average more symptomatic adolescents. Furthermore, the high baseline mean scores both for the BDI-II and the OASIS measures reinforce the determined interpretation that the participants did have substantial mental health symptoms [29, 31]. Previous research has shown that high baseline scores may display rapid symptom improvement [39], but a higher level of mental distress may predict a longer course of treatment [40]. For a number of the participants, the visits were continued, varying from one visit to more than five more visits. Thus, it could be argued, that for the intervention to reach its full potential, treatment should either be offered at an earlier stage to less symptomatic adolescents or alternatively, as a longer intervention.

Additionally, it is noteworthy that the follow-up took place during the COVID-19 pandemic, and we could not control to what extent it, and the restrictions related to the pandemic, may have affected the participants’ symptoms. COVID-19 introduced many more stress-induced factors into the everyday lives of adolescents and thus did contribute to mental health challenges [41].

There are challenges in the current mental health care system for the strictly separated child/adolescent and adult mental health services [42]. Good connections between different settings and supportive interactions among the different actors, such as teachers, parents, community members, and other professionals are indeed relevant for positively promoting the mental well-being of adolescents [16, 17]. Adolescents who show symptoms of mental health distress should be reached through easily accessible interventions at an early stage for the most positive and prominent long-lasting results [12, 43]. By offering that early support, the pressure on specialized mental health care could ease, and ideally more services could be available for adolescents with severe mental symptoms. Also, by rearranging these services, there might be fewer referrals of adolescents to organizations and across different mental health services [12].

There is a great demand for innovative, easy-to-implement methods to improve the accessibility of cost-effective mental health services [15, 38]. To answer this demand, interventions need to be delivered by professionals or school staff who use methods that respond to different types of symptoms to achieve inclusive and easily implement accessible mental health services to all adolescents in school settings [38, 42]. In the present study, although the symptoms relapsed to some extent in the long term, only 23% of the participants needed specialized mental health care. Considering the symptom levels that were learned at baseline, it can be hypothesized that a majority of these individuals would have potentially been remitted to specialized mental health care without an intervention.

This study confirms that early intervention approaches can show prominent results for common adolescent mental disorder symptoms and that school settings are feasible settings for both the identification and the provision of positive interventions for mental health symptoms [39]. Utilizing their earlier skills and short cognitive behavioral therapy training, the psychiatric nurses were able to provide effective treatment for adolescents, yielding benefits that are comparable to treatment given by specialized professionals. However, the nurses do need support to keep up the skills necessary for identifying mental disorders [44]. Furthermore, clear guidelines are still needed on how to proceed if the given help at schools is not sufficient [21]. The low cost for training staff and the easy implementation of intervention that reaches a large span of mental health symptoms could be a solution for providing easily accessible and readily available mental health care to more adolescents in school settings [16]. If needed, that care could be continued with additional booster sessions and possibly even follow-up checks to sustain the mental well-being already achieved.

### Strengths and limitations

The strength of this study is that the treatment was easily accessible for a large span of mental symptoms, and it was delivered in school settings and at a low cost. School health care is accessible and free for all students and makes any treatment less stigmatizing, also equally available and with no economic burden. Also, the psychiatric nurses were able to deliver an effective and structured treatment with only short training. The findings of this study thus need to be viewed considering the limitations noted for the study.

The lack of a control group and small sample size limits the strength of the conclusions*.* To determine the effectiveness and acceptability of this intervention, further research with a control group should be conducted and measures on participant expectations and their adherence to the intervention could be added. Also, to be noted here is that it is still unknown what impacts the COVID-19 pandemic and its restrictions had on the mental well-being of these adolescents during the 6-month follow-up. We focused on the operating model of the intervention and were not able to control for individual factors related to, for example, previous training and the competence of the nurses who were doing the intervention.

## Conclusions

The present study shows that easily accessible intervention in school- settings is associated with improvement for adolescents who have mild to moderate mental disorder symptoms and are attending an upper secondary- or vocational school. These results impacted the general well-being of the adolescents upon immediate follow-up, but the results did not hold at the 6-month follow-up. Yet, even for adolescents with moderately severe symptoms, the intervention still showed positive short-term results. For these results to prevail for those adolescents with moderately severe symptoms, additional visits are needed, or alternatively, the intervention should be offered at an earlier stage. Also, for those adolescents who do need further help, more effective interconnections across health settings are called for [16]. Future studies should thrive to reach those adolescents in school-settings with milder symptoms and at an earlier stage and thereby integrate the comparison groups, while also including longer follow-up assessments.

## Supplementary Information

**Additional file 1.** The TIDier checklist.

## Data Availability

The data that support the findings of this study are not openly available due to reasons of sensitivity but are available from the corresponding author upon reasonable request.

## Abbreviations

CBT: Cognitive behavioral therapy
IPT: Interpersonal therapy
IPC: Interpersonal counselling
YP-CORE: The Young Persons Clinical outcomes for Routine Evaluation
BDI-II: Beck Depression Inventory
OASIS: The Overall Anxiety Severity and Impairment Scale
